# Complete Genome Sequence of NC983, a Live Attenuated Strain of *Salmonella enterica* Serovar Typhimurium

**DOI:** 10.1128/genomeA.01074-16

**Published:** 2016-10-13

**Authors:** Bryan Troxell, Ryan C. Fink, Allison N. Dickey, Elizabeth H. Scholl, Hosni M. Hassan

**Affiliations:** a Prestage Department of Poultry Science, North Carolina State University, Raleigh, North Carolina, USA; b Microbiology Graduate Program, North Carolina State University, Raleigh, North Carolina, USA; c Department of Biology, St. Cloud State University, St. Cloud, Minnesota, USA; d Bioinformatics Research Center, North Carolina State University, Raleigh, North Carolina, USA

## Abstract

Foodborne infections caused by *Salmonella enterica* serovars are a significant problem worldwide. Presented here is the genome sequence of the nontyphoidal *S. enterica* serovar Typhimurium mutant strain NC983, a potential vaccine candidate.

## GENOME ANNOUNCEMENT

Strain NC983 of *Salmonella enterica* serovar Typhimurium is highly attenuated in mice ([1]; H.M. Hassan, unpublished data). NC983 was generated through fusaric acid-mediated removal of the tetracycline marker (2) from an *fnr::*Tn10 mutation in *S*. Typhimurium ATCC 14028s. DNA was extracted from lysed (bead-beaten; Biospec Products, Bartlesville, OK, USA) NC983 cells using a FastDNA SPIN kit for soil (MP Biomedicals, Santa Ana, CA, USA). Eluted DNA was concentrated and processed with the PacBio whole-genome sequencing workflow using the Pacific Biosciences RS II sequencing platform (Pacific Biosciences, Menlo Park, CA, USA). The 20-kb SMRTbell templates kit was used for template preparation. The library was prepared using a 10-kb template library preparation workflow from size-selected templates (BluePippin V3 Cassette Definition for 10,000 bp). Three SMRT cells were used on a PacBio RS II sequencer with the C4 sequencing chemistry and P6 polymerase. The Ion Torrent reads were obtained from fragmented DNA, and libraries were prepared and purified using AMPure beads. Specific adapters Ion P1 and Ion XpressBar code X were ligated to fragmented DNA using the Ion Plus fragment library and barcode adapters kits (Life Technology, Thermo Fisher Division, Waltham, MA, USA). Ligated DNA was nick-repaired, purified, size-selected, and amplified. DNA templates were sequenced on an Ion Torrent PGM using Ion PGM300 sequencing reagents (Life Technology). Base-pair calling and sequence trimming were performed on the Ion Torrent browser.

The PacBio continuous long reads were error-corrected using the Hierarchical Genome Assembly Process (HGAP) workflow (PacBioDevNet; Pacific Biosciences; SMRT Analysis version 2.2), and a *de novo* assembly of the corrected reads was conducted using MIRA version 4.0.2 (3). The resulting assembly (48×) consisted of six contigs, two of which were large nonrepetitive contigs. Ion Torrent reads (19×) were mapped to the alignment of the two large contigs using MIRA to increase the average consensus quality. The resulting consensus contigs were circularized using the Minimus 2 assembler (4) and polished using Quiver (5).

The longer of the two contigs mapped to the chromosomal reference sequence of 14028s (NC_016856.1; 4,870,265 bp), and the shorter of the two contigs mapped to the plasmid reference sequence of 14028s (NC_016855.1; 93,832 bp) (6). Five G/C homopolymer runs in protein-coding regions within the chromosomal contig were corrected by adding either a single G or C to match that of the 14028s reference. After circularization and polishing, the chromosomal DNA was 4,846,304 bp in length (average PacBio base coverage: 298×), and the virulence plasmid, pLST, was 93,829 bp in length (average PacBio base coverage: 461×).

The NCBI Prokaryotic Genome Annotation Pipeline (http://www.ncbi.nlm.nih.gov/genome/annotation_prok) was used for annotation, and it identified 4,612 protein coding-genes with 85 tRNAs, and eight 5S, seven 16S, and seven 23S rRNA genes. Strain NC983 contains a large deletion that removed base pairs 1,737,878 to 1,764,448 from the genome of 14028s (6). This stretch of sequence in 14028s has been replaced in NC983 with a 1,332-bp remnant of the Tn10 transposable element. 

### Accession number(s).

The complete genome and virulence plasmid sequences of NC983 were deposited in GenBank with accession numbers CP015157 and CP015158, respectively.
